# Consumers’ Intention to Adopt Blockchain Food Traceability Technology towards Organic Food Products

**DOI:** 10.3390/ijerph18030912

**Published:** 2021-01-21

**Authors:** Xin Lin, Shu-Chen Chang, Tung-Hsiang Chou, Shih-Chih Chen, Athapol Ruangkanjanases

**Affiliations:** 1 School of Economics and Management, Northeast Electric Power University, Jilin 132012, China; linxin@neepu.edu.cn; 2 College of Management, National Kaohsiung University of Science and Technology, Kaohsiung 824, Taiwan; 0128904@nkust.edu.tw; 3 Department of Information Management, National Kaohsiung University of Science and Technology, Kaohsiung 824, Taiwan; sam@nkust.edu.tw; 4 Chulalongkorn Business School, Chulalongkorn University, Bangkok 10330, Thailand

**Keywords:** food safety, organic food product, information system successful model (ISS), Theory of Planned Behavior (TPB), Trust (TR), Partial Least Squares (PLS), blockchain food traceability system (BFTS)

## Abstract

Establishing a blockchain food traceability system (BFTS) is increasingly important and urgent to resolve the contradiction between consumers’ intention regarding safe food selections and the spread of polluted foods. Using the advantages of blockchain, such as immutability, decentralization, openness, and anonymity, we can build trusted food traceability systems based on these important characteristics. With reliable information, traceability from production to sales can effectively improve food safety. In this research, multiple models, namely, the information success model (ISS) and the Theory of Planned Behavior (TPB) are formed into a conceptual integrated framework to study the intentions’ influenced factors of BFTS technology for Chinese consumers to help ensure food safety and the quality of Chinese organic food products. A face-to-face questionnaire survey with 300 valid responses was analyzed by Partial Least Square from the Chinese consumers focusing on the organic food products. This study found that the attitude and perceived behavioral control qualities significantly and positively affect the usage intention in adopting BFTS, while the subjective norms are positively but not significantly correlation with the usage intention in using BFTS. The above results will inform suggestions for productors and academics along with implications to promote BFTS’ usage intention.

## 1. Introduction

During the last couple of decades, many recurrent food safety issues have troubled people all over the world. According to the statistics released from the World Health Organization (WHO) on 30 April 2020, an estimated 600 million people—almost 10% of the people worldwide—become ill from eating unclean food which directly kills 420 thousand and indirectly costs 33 million deaths each year. In underdeveloped countries, the annual loss of productivity and health care costs due to unsafe food amounts to $110 billion [1].

After eating polluted food, 550 million people fall ill with diarrhea, and 230 thousand people die each year. Unsafe food exacerbates the vicious cycle, formed by disease and malnutrition, particularly the impact on the very young, the elderly and the patients [1]. An essential element of public health is to ensure the safe food. Eating safe, healthy food can provoke our body immunity to fight different diseases, even the COVID-19 outbreak. Food safety is an important factor in keeping fit. Although WHO does not consider eating polluted food to be a cause of COVID-19, there are many opportunities for infection by other foodborne pathogens, which may weaken our body’s immunity [2]. Because of the transmission dynamics of COVID-19, it is thought to be a zoonotic illness [3,4]. Animals are considered to be major repository for coronaviruses, some of which may cause human infection [1,5]. Contently, an unflawed food safety system is also vital to the prevention of the epidemic [5].

Nowadays, under the background of the global food trade, severe information asymmetries exist in the producers and consumers, which prevent consumers from making informed choices when consuming food, leading to a lack of trust in food safety. This has led to several food crises, such as Madcow Disease, African swine fever, Food-and-Mouth Disease, the Europe horsemeat scandal, melamine-contaminated milk powder. Because of the globalization of the market and the problem of safety food quality and environmental protection, consumer demand for food traceability has raised sharply in the past ten years [6]. As a result, the food industry is under increasing pressure to track food production, processing, and sales [7]. Actually, food safety incidents not only seriously jeopardize people’s health and consumer confidence, but also negatively affect the food firms’ profits, the scale of the food industry, and even the nation’s global reputation [6]. All of those factors emphasize the need to implement or improve blockchain food traceability system (BFTS). Furthermore, as an important measure to guarantee food safety, BFTS provides all kinds of necessary information from farm to dish, making up for information asymmetry.

In the 1990s, the Food Safety Commission put forward the food safety record system, named blockchain food traceability system, to decrease the food damage risks and to avoid food-borne cases. Over the past ten years, the EU, the USA, and Japan have all applied blockchain food traceability system (BFTS). In 2015, the Chinese government passed a new Food Safety Law to force food producers to implement BFTS covering the whole process of food production, processing, and circulation [6]. Under the immutable system environment of BFTS, food safety stakeholders can complete the digital traceability of food production process. Traceability information includes supply chain temperature, production date, physical address of transportation stops, country of origin, production conditions, production batch and responsible person. These data are linked with food in the form of digital code, and the unchangeable information of each link will be recorded in the corresponding nodes of the blockchain in turn.

The shortcomings of blockchain technology are mainly reflected in the following aspects: few practical applications of blockchain technology in the food safety industry, more ideas on food safety traceability, fewer implemented measures, and less relevant academic research [7]. Previous research works have studied food safety and BFTS separately from the consumers’ perspective, but few empirical analyses have attempted to relate food safety concerns to the blockchain technology of BFTS. This research fills this gap by studying Chinese consumers’ attention to food safety and the blockchain technology of BFTS [6].

Focusing on China’s traceability system, this research revealed that three types of influences factors on Chinese consumers’ usage intention of organic food products by integrating the Information System Successful Model (ISS) into Theory of Planned Behavior model (TPB), then empirically analyzed the influence of these three factor types on both trust and usage intention. The above empirical analysis results prove that it is beneficial to incorporate these types of theoretical models together. As far as we know, in the field of food safety traceability, there are few empirical studies using the integrated model of ISS model and TPB. This research can fill this knowledge gap.

## 2. Research Background and Literature Review

Currently, the global organic food industry is valued at more than $400 billion, accounting for about 14%of global agricultural trade, and the organic food industry is the third largest agricultural commodity [8].

Organic food refers to food that contains no man-made chemicals such as weedkillers, bactericides, manure, insecticides, and GMOs [9] and is not irradiated. In previous research works, organic food was interpreted in different terms, e.g., local, natural, and unpolluted [9]. The global organic food sales scale has grown rapidly over the last twenty years. In 1999, the sales scale was only $15.2 billion. By 2016, it reached $90 billion and is expected to extend to $320.5 billion in 2025 [10].

### 2.1. Blockchain Food Traceability System (BFTS)

#### 2.1.1. COVID-19 Pandemic

COVID-19 was detected by the end of 2019, and in February 2020, the World Health Organization [11] declared COVID-19 a pandemic. By 1 April 2020, the COVID-19 pandemic broke out in more than 180 countries, causing over 365,000 deaths [12] and affecting at least one-third of the global population’s health [13]. In the COVID-19 epidemic, food safety is a complicated health problem. Obviously, the food safety risks cannot be completely eliminated, but the risks must be controlled throughout the whole supply chain from farmhouse to fork. Reducing safety risks requires close cooperation with departments, investors, and local administration organizations. Commitment among management and decision makers on food safety issues, coordination and partnership among local governments, shareholders and nations, distribution of adequate capitals and accountability from all interested party are necessary to achieve food safety [5].

#### 2.1.2. Organic Food Safety

Food safety refers to the degree of reliability that a food product will not cause illness or injury during the period of being produced, served, or consumed [14]. From the economic point of view, information asymmetry is one of the problems that contribute to food safety. Obviously, suppliers often take advantage of information asymmetries with buyers to participate in opportunistic behavior, for example, fraud. If BFTS can gain buyers’ trust, it will reduce information asymmetry. BFTS has become an important solution for producers to demonstrate the food quality to buyers [15].

Organic agriculture is defined [16] as a production system using natural inputs and specific practices (crop rotation), and forbidding the adoption of pesticides, artificial fertilizers, antibiotic drugs for animals, genetically engineered seeds, and preservatives. Food products move from the farm to the final consumer, through a variety of intermediaries, for example distributors, wholesalers, and retailers, forming a complete agricultural food supply chain. Modern grain production and supply system has beyond regional boundaries and has become a transnational economic operation [16]. Problems in any link of the food chain may affect the whole food chain. Therefore, we must ensure the normal operation of all links of the food chain. This will limit the flow of problems from one stage of the food chain to the next, thus helping to nip the danger in the bud as early as possible [16].

#### 2.1.3. Blockchain Technology

The COVID-19 pandemic has exposed the fragility of traditional supply chains. During such emergencies, most companies around the world find it difficult to continue the flow of their products and services [16]. To solve the situation of lacking a suitable information-shared platform, using a distributed ledger technology, such as blockchain, is a very promising solution [16].

Recently, blockchain has received a lot of research attention in solving food supply chain problems. In 2008, Nakamoto proposed the concept of decentralized point-to-point ledger, which is now generating huge interest in food supply chains, property, voting, and more. In order to improve the information management of agricultural food trade, Wolfert et al. [17] positioned blockchain as the core technology of “network physical management cycle of agri-food production” and developed together with the Internet of Things, big data analysis, artificial intelligence, and other technologies. Blockchain’s underlying structure has several key characteristics that, when used properly, will provide vital advantages: decentralized organization, fixity, safety, and smart contract. Being unchallengeable and transparent, blockchain with distributed ledger characteristics provides a secure, fast, and trusted solutions. Transactions stored on the blockchain are treated as records in blocks. The block also contains a time stamp and a hash value that connects it to the previous block to form a block chain that cannot be changed. 

The characteristics of blockchain are as follows:

(1) Immutableness: Due to the existence of immutable characteristics such as cryptography, Hash function, and miner calculation, blockchain technology ensures block a highly trusted Internet environment can be built. Since tampering with a blockchain record means tampering with millions of other instance nodes on this chain at the same time, the information on the blockchain can only be added and cannot be replaced, the record will be permanently and authentically recorded, and every transaction record on the blockchain is immutable [18].
(2) Decentralization: Data storage in blockchain is distributed over every node of the network, with a high degree of autonomy. Unlike traditional storage methods, it does not rely on a special trustworthy center system consisting of one or several larger nodes, all nodes are involved in the validation, storage, and preservation of each blockchain information, which is named decentralization [18].
(3) Openness: Blockchain makes the necessary data on the chain open to anyone through a consensus mechanism. All trading parties can use the timestamp mechanism to trace the information of goods, which increases the trust between buyers and sellers. In addition, it also facilitates the monitoring and epidemic prevention and control by government agencies. In short, it can help establish a highly transparent food safety traceability mechanism [18].
(4) Anonymity: The anonymity of blockchains means that the transaction process can hide real names. The privacy of blockchain trading nodes and the personal information of users can be hidden by cryptography. This means using blockchain can trace the product data information part of blockchain transactions, but cryptography can protect the identity and privacy of consumers, thus effectively preventing the disclosure of personal and private information [18].

Generally, food traceability is the recording of information related to the various stages of food from raw materials, soil, tillage or growing, harvesting, processing, manufacturing, transportation, and distribution. For safety and health reasons, it is necessary to declare the place of origin, time of delivery, and problem bearers. Consumers scan barcodes to capture data during production, processing, and delivery. A standard food product information list includes IOT number, validation mark, manufacturer, termination date and barcode. Transparent food information improves the effect of food traceability, and boosts consumer trust and motivates them to buy trustworthy products [19].

#### 2.1.4. Blockchain Food Traceability System

The need for food safety plasticity is even more urgent considering that COVID-19 is already detected in food, on surfaces and in the surrounding environment. At present, there is no evidence that coronavirus can be transmitted through food, so no research has been carried out or a method has been developed to detect the virus in food [20]. However, as noted above, infected workers have the potential to spread infection through the food industry and through the environment of the food supply chain [21]. Some companies have developed commercial kits or surface sampling kits for detecting COVID-19 in environmental swabs. However, sampling kits in particular are so expensive that large sampling facilities, especially in the food sector, can hardly be used widely [2].

The blockchain food traceability system, defined as part of logistics management as an information system that captures, stores, and transmits information about collection, rearing, and production at all stages of the food supply chain, and blockchain food traceability systems, allow consumers to receive any required food safety and food quality control checks, as well as backup data information to better serve the food safety needs of consumers. Ringsberg [15] also argues that traceability is the process of finding out the means of determining the cause of defects in food safety failures in the supply chain [22]. BFTS is considered as a major tool to eliminate information asymmetry and fundamentally prevent the potential food safety incidents [23].

As a blockchain food traceability system identification tool, barcodes have been combined with BFTS to track food products fast and accurately. The traceability labels are always printed with the Quick Response (QR) code as a two-dimensional bar code [24]. A QR code can store adequate data and has very good readability. It is also very readable when part of the code is physically damaged [25]. In advanced countries, QR codes are considered to be an upgraded version of linear bar codes because they provide higher data storage and encryption capabilities, as well as environmental protection [26]. Compared with RFID and traditional bar codes, one advantage of two-dimensional code is that it can match a variety of two-dimensional code decoding software and systems, rather than limited to reader devices, so it can be decoded by a variety of devices including mobile phones [8]. According to the description of data flow and blockchain flow in the logistics process, the BFTS records all kinds of information under and on the chain at different stages of the food trading chain. The client can load the system with a QR code on the food label to access the main information related to food traceability [27].

### 2.2. ISS Model

ISS is a model for measuring the success of information systems proposed by DeLone and McLean [28] that has been widely used since its publication. In 2002, considering of the empirical test results and theoretical discussions of scholars, they revised their model to measure information success techniques [28]. System quality (SYQ), information quality (IQ), and service quality (SEQ)are all the vital affecting elements, which finally influence usage intention by user satisfaction [29]. As the most popular IS framework to measure the success of information system techniques, it has been widely recognized and applied in various research areas. At present, ISS has been utilized to evaluate the success degree by more than 280 articles [30]. In particular, ISS has been utilized to many aspects of information system, for example, mobile payment [31], mobile banking, and mobile learning [32].

Although ISS [28] is a compelling model for predicting technology utilization, the purpose of usage intention is to focus on a user’s task rather than on transactional activities. Under the background of food safety, customers use BFTS to make purchase decisions and execute commercial transactions. Unless BFTS is reliable enough, the decision is risky. Therefore, this study suggests that the initial trust theory should be used to strengthen customer’s purchase intention [29].

### 2.3. Theory of Planned Behavior Model (TPB)

TPB [33] has been applied as the conceptual framework for this research, and has been proven successful in predicting consumers’ usage intention in choosing safe food [34]. TPB is composed of ATT, SN, and PBC. TPB assumes attitudes (ATT; positive or negative evaluation behavior), subjective norms (SN; influence of other people’s thoughts and attitudes toward behavior), and perceived behavioral control (PBC; the degree to which an individual feels capable of performing an action) result in the formation of behavioral intention that will facilitate the willingness to take an action. In short, behavioral beliefs generate ATT, normative beliefs generate SN, and controlling beliefs generate PBC. All these factors combine to form intention.

TPB has been applied to study the vital factors which can influence usage intention of consumers’ food-decision in many fields: food hygiene interventions, the genetical- modified agricultural, and public perception’s indicators on food additives [35]. As a tool to analyze the relationship between food-choice behaviors risk-related behaviors, TPB is rather practical in predicting people’s food-consumed decision patterns or food processing methods [36]. TPB is an important social psychological theory for predicting consumers’ behaviors [36]. ATT is a consumer’s overall evaluation of target behavior. The attitude towards a particular behavior reveals the actor’s trust in that behavior’s implications. Ajzen [33,37] believes that the attitude towards a certain technology should be measured by trust, which means the actor’s tendency to trust the target behavior.

### 2.4. Trust (TR)

Trust is a person’s willingness to rely on a new technology or a person’s will [38]. Thus, trust ensures that users are empowered to make decisions in the face of uncertain evidence of reliability, and thus to purchase sustainable, healthy, truthful, and safe food according to their own use intentions [39].

Consumers want transparency and effective accountability in the food production chain using information technology to understand sources and processes from farm to fork, ultimately increasing trust in food safety. The lack of reliable information about transactions in the marketplace, such as dishonest and deceptive practices, may lead to a failure to gain consumer trust, whereas the provision of credible food information can greatly enhance consumer trust [40].

## 3. Research Model

There is a lot of evidence pointing out that ISS and TPB can systematically clarify and predict the factors affecting the use intention of information system, respectively. However, although TPB has been successful in predicting usage intention, usage intention and behaviors in many areas, it cannot disclose every component of a particular intention, i.e., food choice decisions [36]. TPB should be extended with some extra components to better reveal usage intention.

**Figure 1:**
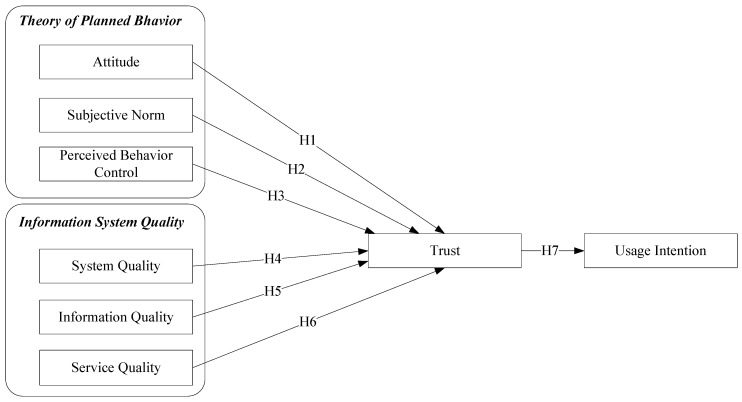
Research model.

There are few empirical reports on food safety when TPB are combined into ISS. According to Figure 1 of this research, in view of the deficiencies of theoretical structure and empirical analysis of food safety quick inspection, two series of factors (namely ISS model and TPB model) are combined to form a complete comprehensive model as the conceptual framework. The conceptual framework makes up for the interpretation defects of two independent models and further clarifies the subjective and objective factors that influence the intention of transformation. After filling the above gaps, this study will comprehensively understand the influencing factors of organic food consumers’ willingness to use, contribute to the design of traceability system for organic food products and services, and further expand the benefit scale of organic food enterprises.

### 3.1. TPB Model

The previous research works have approved the effect of attitudes on usage intention [35]. ATT has several types, for example, attitude toward buying traceable ground beef/steak were measured on four scales of semantic difference compared with those available in stores: affective (bad-good, unpleasant-happy) attitudes and cognitive (stupid-wise, harmful-beneficial) attitudes [41].

**Hypothesis** **1** **(H1).**  *Attitude significantly influences trust towards BFTS.*

Subjective norms (SN) are prerequisites for the implementation of social behavioral intentions, including the IT systems [35,42]. Perceptions of social pressure to buy traceable products fall into five categories of social norms: family and friends, university scientists, the media, the food industry, and other important people [38]. SN enables consumers to naturally change their intentions according to certain norms or opinions of the majority [42]. Numerous studies have shown that peremptory and descriptive norms have an active influence on usage intention. The greater the influence of a person or organization on the public when they are faced with reporting dilemmas, the greater the pressure on them to participate in food safety monitoring, thereby increasing their intention to participate [35].

**Hypothesis** **2** **(H2).**  *Subjective norm significantly influences trust towards BFTS.*

PBC mentions people’s cognition of the difficulty in carrying out a certain interest behavior [34], or the degree to which an individual perceives that the behavior is controlled by his or her intentions [43]. PBC determines how an individual can perform a behavior, and it can influence the trust, as well as indirectly affect the intention through trust. Public food safety reporting behavior can be affected by many objective factors [35]. To measure usage intention, participants rated traceable food as more likely be healthier, more expensive, of known origin, of better quality, truer, more environmentally friendly, with higher animal welfare standards) [41]. Given the above situation, we can hypothesize that:

**Hypothesis** **3** **(H3).**  *Perceived behavior control significantly influences trust towards BFTS.*

### 3.2. D&M ISS Model

SYQ, IQ, and SEQ are designed to provide belief and stimulate trust in wireless business. The definition of system quality is to measure the degree of the user’s perceived intensity, the degree of pleasure in use, adaptability, and response time that are esteemed by users of a wireless system [29]. Vance et al. [44] also pointed out that SYQ influences users’ trust in wireless technologies [45]. Chung [33] researched the Korean mobile service access terminal convenience, system reliability, response time, and flexibility and other influencing factors, in order to determine how SYQ becomes the key variable of service loyalty [46]. Sharma [34] pointed out that, without the above functions, consumers may doubt the ability of mobile service providers, thus increasing the difficulty of using the equipment [47].

A user-friendly interface means navigation ease of use, response time, and reliability. Therefore, we hypothesized that system quality is a key factor determining trust and has a significant positive relationship with trust. Therefore, H4 is established:

**Hypothesis** **4** **(H4).**  *System quality significantly influences trust towards BFTS.*

DeLone and McLean confirmed that the characteristics of IQ, for example, completeness, accuracy, relevance, and accessibility, are the basic elements of usability, and pointed out that IQ is an important factor determining consumers’ intention to use a certain technology [32]. IQ is used to measure the quality of the wireless service information. The information about the wireless technology should be personalized, complete, relevant, easy to understand, so that customers can be risk-free when engaging in any interactive business. Therefore, IQ must be considered as a core construction affecting relationship quality and switching intention [29].

Jeong and Lambert [35] believed that in an environment with FTS as the intermediary, customers’ switching intention of some firm’s products and services can be determined by their IQ [48]. Studies have also shown that the dairy products’ IQ is an important part of building a positive reputation [28]. IQ refers to the quality of system output products, including relevance, user-friendliness, adequacy, and accuracy [49]. Therefore, the hypothesis is suggested as the following:

**Hypothesis** **5** **(H5).**  *Information quality significantly influences trust towards BFTS.*

SEQ refers to the service qualities (such as responsiveness, trustworthiness, simplicity, and technological ability) that users can get from the information systems departments, or others. Only when service quality is concerned can the effect of information system be evaluated correctly. We trust that identifying all quality elements, including SEQ, will help consumers correctly establish and measure the relationship quality [49].

SEQ is the measurement of the whole support provided by a wireless service provider. Service quality plays an important and positive role in trust. The ability to complete wireless business, integrity, after-sale service, and their understanding of customer needs are the most critical factors in SEQ [29].

SEQ also affects users’ satisfaction, trust, and service transformation. For example, customers who evaluate high SEQ show positive intentions; on the contrary, customers with low SEQ are more likely to change products rather than buy the same products in the next time when they face the same choice situation [48]. Thus, H6 is established.

**Hypothesis** **6** **(H6).**  *Service quality significantly influences trust towards BFTS.*

### 3.3. Trust (TR)

Trust (TR) refers to the intentional situation in which an individual believes that future behavior will follow appropriate actions of reliability and competence [50,51]. This study takes the continued willingness in using BFTS during the COVID-19 pandemic as an object, trust can significantly shape users’ psychological expectations to believe that BFTS can provide reliable services [52], that is, the higher the accumulation of trust in BFTS, the higher willingness to continue to use BFTS.

Trust in food safety traceability systems at all levels, from producers to government regulators to consumers, is extremely important and enhances consumers’ willingness to use food safety [53]. Trust in food safety traceability refers to trust in safe food, as well as the prediction of loyalty of the main actors providing the safety food [54], also predicting loyalty [55]. Conversely, unsafe food incidents can only increase risk and reduce the trust [56].

At the same time, trust was pointed out to be a significant variable of users’ willingness to continue using mobile technology [57,58]. Additionally, trust was also proven to be an important positive factor in mobile technology acceptance, such as mobile banking [59], mobile websites [60], etc. Thus, by using trust as an additional ISS variable, the hypothesis is that:

**Hypothesis** **7** **(H7).**  *Trust significantly influences usage intention towards BFTS.*

## 4. Data Collection and Results

In August 2018, the first Fresh Hema store of Alibaba in Beijing, China, opened, covering nine categories of fresh products, such as meat, vegetables, eggs, milk, fruits, and aquatic products, nearly 1700 “box horse day fresh” products realized the whole chain dynamic traceability, and led more than 2 million users to scan the code to check the traceability information of products, effectively boosting consumers’ confidence in food safety. This time, with its advantage of scale effect, Fresh Hema found the organic source areas for direct mining, predicted the order quantity according to its own digital ability and existing user demand, and achieved the goal of accurate supply and reducing loss. Together with Alibaba cloud, Fresh Hema builds “IOT intelligent vegetable base” and “Internet of things+agriculture” covers all aspects of tracking pre prevention, in-process control, and post tracking. By using these grippers, the supply chain can be upgraded and digitized, visualized, and traceable, to ensure the best quality, fastest transportation and minimum consumption of vegetables.

From 9 October to 9 November 2020, a 4-week period of face-to-face interview was conducted on the factors influencing the willingness to use the organic food traceability system in Fresh Hema store’s organic food products’ BFTS in Dalian City, Liaoning Province, China. First, a survey questionnaire was designed based on the seven hypotheses above. In the pre-testing stage, according to the theme of this study and the background of epidemic prevention and control, the relevant measurement items of most variables were improved by referring to the latest literatures. Through face-to-face interviews, we invited about 50 participants who often used the Fresh Hema APP (including the related BFTS system) for trading were invited to complete the pre-test. Then, the measurement items that were not targeted or easily misunderstood in the questionnaire were strengthened and improved, so that the participants could fully understand the meaning of the items and upgrade the accuracy of the questionnaire. In the second round, about 300 respondents were surveyed. After excluding those who had never used Hema Xiansheng’s BFTS, relevant data were obtained from senior Hema Xiansheng users through face-to-face interviews. Because the Hema fresh store in Dalian operates better than the rest of northeast China, enough respondents were quickly interviewed.

We issued 350 questionnaires of which 320 were recovered, with a response rate of 91.42%. A total of 300 samples (93.75%) were used for deterministic analysis after 20 answers were given up. The selected data was appropriate to determine the sample. The project was provided with a five-point Likert scale (Table 1), varying between “strongly rejected” and “strongly agreed”.

In our research, the two-stage method recommended by Anderson et al. [61] was used to evaluate the external model of each variable and to examine the internal model and related hypotheses. Cronbach’s approach was applied to judge the effectiveness of the structure, and the factor structure and internal correlation of each structure were investigated. To test the study hypothesis, we used SmartPLS 3.2.9 (Boenningstedt: SmartPLS GmbH, Boenningstedt, Germany) to determine causality by means of significance values and standard coefficients. Before hypothesis testing, the combined model was studied by all samples. In order to verify the hypothesis, we made a more specific analysis of the models applied in each group.

### 4.1. Reliability, Validity and Measurement Model Evaluation

Convergent validity is the intensity of correlation between variables directly tested in a construct. The correlation is directly proportional to convergent validity. The following criteria should be considered, according to Fornell and Larcker [62]:

- Standardized loadings of every model must be greater than 0.7;
- Composite reliability must be greater than 0.6;
- Average Variance Extracted (AVE: measures the relationship between the amount of variance captured by a construct and the amount of variance caused by measurement errors) must be greater than 0.5.

We evaluated the convergence effect of the measurement items on its related structures. First and foremost, the reliability was measured by standard factor loadings. Moreover, reliability measurement was evaluated by Cronbach’s α and CR scale. For example, composite reliability was used to evaluate internal consistency. Table 2 shows that CR values are all over 0.7, indicating that internal consistency is acceptable. Furthermore, AVE is extracted to measure the variance of a variable relative to the variance.

As shown in Table 2, every standardized loading of each model is above 0.7, which meet the criterion for next analysis. Additionally, Cronbach’s α (as shown in Table 2) is better than 0.60, and CR is far higher than 0.6 [63], showing that the best validity measurement explains the structure of the scale and the overall consistency level is high. Summarily, Table 2 shows that the standardized loadings, CR, and AVE are all higher than the numerical values suggested by Fornell and Laracker [62]. Therefore, all constructs exhibit good convergent validity [62].

According to Table 3, discriminant validity is the difference between the correlation index of the first principle and the correlation index of the second principle [64]. Fornell and Larcker [62] found that when conducting discriminant validity test, the square root of AVE in the correlation coefficient of each construct of each variable must be evaluated. 

In Table 3, discriminant validity is mainly used to test the differences of every structure under external mode. As can be seen from Table 3, for each data, the square root of the variance between each structure and each AVE is greater than any correlation coefficient between the relationship structure between the structure and the other structure, which satisfies a good criterion of discriminant validity. The correlation coefficients between the constructs exceeded the diagonal values, demonstrating that the construct validity of our measurement tool was satisfactory.

SEM tests were performed on the seven hypotheses of this study. For the shrinkage fit indices, the minimum acceptable fit value was exceeded as the standard value. The fit indices indicate that the fit of the analytical sample and the combined model is satisfactory.

### 4.2. Hypothesis Verification

**Figure 2:**
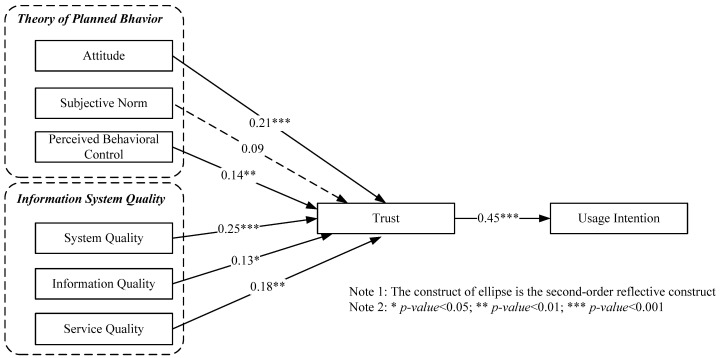
Path analysis of the model.

The path coefficient can be used to study the possible causal relationship between statistical variables in structural equation modeling. We multiply the ordinary regression coefficients by the standard deviations of the corresponding explanatory variables: these standard deviations are then compared to assess the relative effects of the variables in the fitted regression model. After investigating the suitability of measurement and the organization of the integrated framework, the path coefficient of the structure was estimated. Based on *p*-value (Figure 2), one path (H2; *p*-value = 0.097 > 0.05) was rejected and the remaining six paths were confirmed positively.

The consumers’ usage intention was influenced by ATT (β = 0.210), SN (β = 0.089), PBC (β = 0.136), SYQ (β = 2.51), IQ (β = 0.130), SEQ (β = 0.188), TR (β = 0.447), jointly explained 43.4% usage intention.

Table 4 and Figure 2 show that attitude has a significant positive effect on trust, supporting H1 (ATT→TR: β = 0.210, *T*-value = 3.779). However, the subjective norm has no significant direct effect on trust, and therefore, H2 is not supported (SN→TR: β = 0.089, *T*-value = 1.661). The analysis showed that PBC and PE had a significant effect on TR, supporting H3 and H4 (PBC→TR: β = 0.136, *T*-value = 2.610; SYQ→TR: β = 2.51, *T*-value = 4.678). H5 and H6 were also supported in this study (IQ→TR; β = 0.130, *T*-value = 2.181; SEQ→TR; β = 0.188, *T*-value = 3.208). Finally, the results of the analysis showed that TR had a positive and significant effect on SWI (TR→UI: β = 0.447, *T*-value = 9.483). This indicates that our proposed model has sufficient predictive power.

## 5. Discussion

This study shows that there are a number of incentives or drivers associated with food safety issues (food fraud, unsafe product recall, epidemic prevention and control); quality systems (quality assurance systems); perceived behavior control (safe behavior and behavior quality requirements); cost reduction (target recall, efficiency); openness (high transparency, ease of operation); subjective norms (consumer feedback, commodity disputes); immutability (immutability of specific food information); competitive advantage (consumer trust, blockchain traceability technology development, brand differentiation) [65,66]. Under the COVID-19 epidemic, blockchain traceability system can significantly affect consumer trust, enhance purchase motivation, and provide a competitive advantage [67].

In this research, we focus on the elements which can promote consumers’ intention to increase or decrease the adaptation of BFTS. Thus, we took the combination of TPB and ISS as the premise of the framework. Particularly, we identified the important elements that affect usage intention. This study shows that all the other 6 hypotheses are valid except SN.

Our research reveals the following results:

(1) H1 was confirmed that optimistic attitude towards BFTS system of organic food can significantly affect consumer trust, consistent with previous findings [68]. Judged by the results related to H1 (β = 0.210; *p* < 0.001), ATT is a significant element of trust. Our results support H1 that positive ATT towards BFTS of organic predicted trust, which it is consistent with previous studies on organic food [69,70,71].
(2) H2 was unsupported because no evidence can confirm the relationships between SN and trust [72], and does not agree with the previous studies [73]. According to the results regarding H2 (β = 0.089; *p* > 0.05), there were no relationships between SN and trust for the BFTS of organic food (i.e., no support for H2), This result supports H2 but does not support the previous research [73,74,75,76].
(3) H3 was supported by our proposed model, and PBC may positively influence trust (β = 0.136, *p* <0.001). PBC’s significant path to intention confirmed H3, in line with previous studies [68,77]. The current survey results showed that Chinese consumers’ intentions to buy organic food were best explained by the perception of PBC over the purchase of organic food. H3 confirmed by the significant path, is similar with previous studies [75,77].
(4) H4 (β = 2.51; *p* < 0.001) about system quality’s effect on trust was supported: our study showed that SYQ is a vital element of consumers’ trust. SYQ projects reflect access speed, ease of use, navigation, and visual appeal. According to the previous literature [30], when BFTS was designed, system response time, ease of navigation, reliability, and the quality of the layout of the interface are all credibility factors, leading to the establishment of trust.
(5) H5 (β = 0.130; *p* < 0.05) about the influence of information quality on trust was supported: IQ was revealed to have positive effect on trust. As we proposed model explains: IQ significantly influence trust, which supports the H5 (𝛽 = 0.13, *p* < 0.05). The results are similar to those of previous researches who have shown that security, privacy, relevancy, and integrity play important role in developing trust [30,78]. The empirical results of this study show that information quality has a significant impact on trust. Clearly providing complete, accurate and up-to-date product intelligence is critical to maintaining high customer trust. Due to the inevitable errors in the process of BTFS providing relevant product information, low quality product intelligence information will damage users’ trust in BTFS providers to some extent.
(6) H6 (β = 0.188; *p* < 0.01) about service quality was supported: our research exposed that SEQ was another vital element of trust. If SEQ provided by BFTS providers can satisfy customers, and customers’ trust can be cultivated. Numerous previous researches’ results have confirmed that SEQ was extremely important to the consumers’ trust belief [79,80]. Among the elements that influence trust, SEQ has a greater influence (β = 0.18, *p* = 0.001). To provide quality services for users, BFTS suppliers need constant technological iteration and resource investment [6]. Clearly, SEQ can serve as a typical trust “barometer” index. If the reliability, timeliness, and personalization of BFTS cannot reach high enough quality, users will doubt the ability of service providers, which will lead to the decline of trust. We suggest that BFTS providers take advantage of the digital encryption capabilities of blockchain to ensure the safety of organic food. Avoid consumers turning away from BTFS technology because of the huge potential risks associated with it.
(7) Supported by some previous researches, H7 (β = 0.447; *p* < 0.001) was confirmed: for example, Suh and Han [81] revealed that trust acted as an intermediary between perception of behavior control and usage intention. In some empirical studies have also revealed that the level of trust positively influenced the intention to accept the technology [78,82,83]. McKnight et al. [84,85] showed a close relationship between trust and usage intention.

In addition, our integration model reveals that trust is the main factor in determining the use of BFTS to enhance the purchase intention of machine products. It is in consistent with other researches who have confirmed that trust is a vital motivational element in the management process [86,87]. More importantly, our findings show that trust in BFTS is the only trust type exerts an outstanding predictive ability on intention to purchase organic products. H8, which is supported by the above analysis results, is in consistent with the previous studies [54,86,87,88,89].

## 6. Conclusions

### 6.1. Theoretical Contribution

The main contribution of this study is that attempts to evaluate the influencing factors of belief on the willingness to use the food safety BFTS system through the empirical analysis of multiple models. The results confirm that trust significantly influence the willingness of food consumers to use BFTS, and the TPB model and ISS model play a vital role in the cultivation of trust cognition, and customers’ shopping intentions will be stimulated accordingly.

Firstly, most previous studies have focused on traceability up to the retail in the food chain, so there has been a lack of tracing of the consumer part. The consumer willingness component is also important in food safety, and the costs to governments, consumers, individual companies, and the food industry can be severe if such measures are not taken, or if the BFTS system is inefficient. In short, if market forces, consumer demand, and government regulation all take supply chain visibility to a new level, the traceability of food from farm to fork will become a reality. Therefore, in our research, the major role of trust in the organic food supply system provider is primarily about trust in the organic products produced. Thus, we are referring to the trust in the integrity of the entire range of organic foods available on the market.

Secondly, the conclusion can systematically and empirically explain the main elements affecting the usage intention. This study constructed a complete multi-dimensional framework of food safety BFTS. It is proposed that SYQ, IQ, SEQ, TR, AT, SN, PBC, SAT, and TR, which are ten important determinants and factors to measure the usage intention of BFTS.

Thirdly, our case study refers to the blockchain technology of the organic’s BFTS, but there is no doubt that the same approach applies to any area of industrial and agricultural products, as long as the process of using the blockchain technology in this area generates value. Overall, through empirical analysis, this paper first clarified the relationship between consumers’ trust in BFTS and consumers’ willingness to use the food traceability system. Although it is very important to analyze the influencing factors of consumers’ willingness to use food traceability system for the promotion of BFTS, in order to ensure food safety during COVID-19, it is necessary to verify the practice of BFTS.

### 6.2. Managerial Implications

Given the vulnerability of the livestock value chains (including organic food products), immediate action is needed by the Chinese government to ensure the survival of the livestock sector during the COVID-19 pandemic and to restore the livelihoods of livestock and poultry breeders. Manufacturers should use BFTS to target various influencing factors, improve the public’s willingness to use safe organic food products, and publicize the nutritional significance of animal-derived foods (meat, eggs, milk), to improve the overall health and immunity of the nation, and vigorously publicize that livestock and poultry are not related to the transmission of COVID-19 [5].

By using more accurate BFTS, the food industry can promote the provision of information to end consumers and take proactive actions to increase consumer trust and loyalty [14]. BFTS’s strengths include ensuring the safety and quality of organic products and improving the overall effectiveness of the food supply chain by monitoring potentially threatening food safety information. Traceability systems can also help diagnose problems and provide information about the organic food supply chain to upstream authorities and downstream customers. In addition, traceability systems help identify the flow of potentially unsafe products so that preventive actions can be taken in a timely manner.

The managerial implications of this research are expressed in the following recommendations:

Firstly, during the COVID-19 epidemic, the government should play a pivotal role in ensuring that safe food production is fully considered and founded by promoting BFTS’ development. Once the occurrence of a food safety incident, the government and relevant regulatory authorities should promptly start the emergency plan, control the development of the situation, and remove, recall, and seal the problem products. At the same time, manufacturers should also explain the truth to consumers, and put forward remedial measures, and implement them, in order to eliminate consumer panic, enhance consumer confidence. At the same time, without a sound food safety blockchain traceability system, it would have been difficult to trace the origin of meat and vegetables to be identified in the event of food safety problems, so it is necessary for the Chinese government to implement a nationwide information system for a food safety blockchain traceability system. This requires the implementation of a unified collection standard, code rules, transmission mode, port specification, and traceability process in the pilot cities, to ensure timely and accurate information communication between all links of the supply chain and between different traceability technology platforms. In the initial stage of construction, we can focus on urban wholesale markets, large chain supermarkets and designated livestock slaughterhouses. Through the demonstration effect of the blockchain circulation traceability system for organic meat and organic vegetables, China will establish a blockchain traceability security system for traditional tea products, organic milk powder and aquatic products in different regions.

Secondly, BFTS can help food producers know what the consumers’ necessary safety food. BFTS can also play a role in bringing together different actors and departments in the food supply chain to ensure that everyone is aware of the importance of food safety. BFTS provides accountability for transparency in the sourcing and processing of food. Organic food safety management includes the monitoring of organic ingredients, classification of uses, and oversight of final commodity safety. The model in this paper comprehensively measures consumer trust in the BFTS and relates it to consumer confidence in the use of food safety. We extend the existing theoretical framework by measuring factors influencing trust in the BFTS and confidence in the integrity of the BFTS technology used in food production and manufacturing. In the traditional supply chain, each stakeholder of organic food can only have a small part of the information of a certain link of a single finished product. Therefore, it involves a very high cost of time and effort to fully track all the information of a product in the whole supply chain [88]. As a distributed ledger, blockchain allows operators to transmit every information record in the transaction process, which will be stored in a shared block node. Since each shared block node has a corresponding timestamp, the blockchain timestamp will constitute a transaction history chain. During the COVID-19 epidemic, users at each node in the organic food supply chain were able to obtain traceable product information through standard digital port information scanning, which was very effective in dealing with issues such as organic food contamination in a timely manner.

Thirdly, the main elements derived from our research’s results will help the food industry to speed up the construction of digital blockchain food traceability system and improve the efficiency, thereby strengthening the systematic vulnerability of food safety and the prevention of problematic food by using high-tech technologies. Theoretically, consumers’ tracing of blockchain history can effectively lock the source of the problem. Meanwhile, according to the “real-time” location of products provided by the blockchain tracing system, the government food safety department can also clearly grasp the real-time distribution of the problem products among customers in different regions. In this way, there is no need to recall all the products in the whole supply chain, but only recall the contaminated batches of products, to minimize the recall cost while controlling the contaminated food to the greatest extent. In fact, food safety traceability blockchain system that can provide real data can also achieve more effective food safety control. For example, when the distributor receives the goods to be distributed, the distribution link information is immediately stored to the corresponding block information node of the blockchain, a process that truthfully reflects the fact that the manufacturer no longer owns the batch of food. Next, when the carrier will deliver the goods and the customer will acknowledge receipt of the goods, this information will be recorded in the blockchain ledger, which will be considered as the goods are now in the customer’s hands.

Fourthly, in recent years, the emergence of emerging technologies such as the Internet of Things, cloud computing, 5G, blockchain and artificial intelligence has aroused the interest of academia and industry. Recently, a great deal of research has been conducted around the world to adapt these technologies to different fields and to further develop them. Artificial intelligence (AI) and machine learning (ML), in particular, have become the major technologies entering all key areas, including logistics and supply chains. The main reason for the successful system of Artificial intelligence in blockchain is its potential to simplify complex supply chain processes. McKinsey estimates that the use of ARTIFICIAL intelligence in the blockchain could help companies achieve up to us $2 trillion in economic benefits per year [16].

### 6.3. Limitations and Future Work

In the short term, it is hard to ascertain the exact impact of COVID-19 on people, the world economy and food system, and the organic food industry products. However, significant changes in food traceability management methods will consolidate the progress that countries have made in food safety and food security over the past few decades. There are still three limitations deserves further research: first, but future study directions remain to be discovered.

Firstly, future research could include factors such as consumer country and age as moderators in the food traceability integration model, and it is necessary to use this to conduct follow-up studies in order to examine whether there are differences between different consumer samples. Although a food safety traceability framework based on the concept of BFTS has been proposed, new issues concerning trust have also emerged, which should be given attention in future studies, such as the credibility of food safety data, trust based on intelligent contract, and the consensus mechanism in the trust culture. In the follow-up studies, qualitative and quantitative measures will be taken to determine which factors have a positive impact on trust and the intention to use organic food. This will be conducive to the further development of blockchain systems in safe food trading.

Secondly, we only studied the BFTS in the two-dimensional code technology field. The system of the Internet of Things in the food Supply chain is very promising. After all, the Internet of Things covers everything from precision agriculture to food production, processing, storage, distribution, and consumption, which is called from farm to table. The solutions of Internet of Things (IOT) have a broad potential to solve the challenges of traceability, visibility, and controllability. In the near future, a safer, more efficient, and more sustainable BFTS is to be expected.

Thirdly, we combined ISS with TPB to identify the elements that affect the willingness to use BFTS. Future studies could use ISS, social media theory, and so on to test the role of usage intention. Future SEM research should test the usage intention of BFTS in the field of food safety from a broader view.

## Figures and Tables

**Table 1:** Sample characteristics.

Division		Numbers	Percentage
Gender	Male	83	27.67%
	Female	217	72.33%
Age	Below 30	200	66.67%
	30–40	45	15.00%
	40–50	40	13.33%
	Above 50	15	0.05%
Occupation	Company employee	150	50.00%
	Civil servant	85	28.33%
	Self-employed person	40	13.33%
	Others	25	8.34%

**Table 2:** convergent validity and reliability (Entire Samples).

Construct	Indicators	Standardized Loading	Cronbach’s α	Composite Reliability	AVE
ATT	ATT1-4	0.873–0.885	0.896	0.928	0.762
SN	ISN1-4	0.795–0.858	0.856	0.902	0.697
PBC	PBC1-4	0.822–0.883	0.881	0.918	0.737
SYQ	SYQ1-4	0.776–0.898	0.862	0.904	0.703
IQ	IQ1-4	0.811–0.878	0.873	0.912	0.722
SEQ	SEQ1-4	0.795–0.904	0.870	0.910	0.718
TR	TR1-4	0.899–0.927	0.904	0.923	0.599
UI	UI1-4	0.890–0.920	0.926	0.948	0.819

**Table 3:** Discriminant validity (entire sample).

	AT	IQ	PBC	TR	SEQ	SN	UI	SYQ
ATT	0.873							
IQ	0.251	0.850						
PBC	0.489	0.122	0.858					
TR	0.305	0.111	0.238	0.774				
SEQ	0.292	0.174	0.168	0.156	0.847			
SN	0.524	0.228	0.395	0.254	0.247	0.835		
UI	0.460	0.173	0.380	0.566	0.329	0.367	0.905	
SYQ	−0.019	−0.209	−0.064	0.183	−0.217	−0.040	0.058	0.838

**Table 4:** Results of hypotheses tests.

Hypothesis	Route	Path Coefficients	S.E.	*T*-Value	*p*
H1	ATT→TR	0.210 ***	0.056	3.779	0.000
H2	SN→TR	0.089	0.054	1.661	0.097
H3	PBC→TR	0.136 **	0.052	2.610	0.009
H4	SYQ→TR	0.251 ***	0.054	4.678	0.000
H5	IQ→TR	0.130 *	0.06	2.181	0.029
H6	SEQ→TR	0.188 **	0.059	3.208	0.001
H7	TR→UI	0.447 ***	0.047	9.483	0.000

Note: * *p*-value < 0.05; ** *p*-value < 0.01; *** *p*-value < 0.001.

## Data Availability

The raw data supporting the conclusions of this article will be made available by the authors, without undue reservation, to any qualified researchers.
